# A lysosomal K^+^ channel regulates large particle phagocytosis by facilitating lysosome Ca^2+^ release

**DOI:** 10.1038/s41598-020-57874-2

**Published:** 2020-01-23

**Authors:** Xue Sun, Mengnan Xu, Qi Cao, Peng Huang, Xiaojuan Zhu, Xian-Ping Dong

**Affiliations:** 1 Key Laboratory of Molecular Epigenetics of Ministry of Education, Institute of Cytology and Genetics, Northeast Normal University, Changchun, Jilin China; 2 Department of Physiology and Biophysics, Dalhousie University, Sir Charles Tupper Medical Building, 5850 College Street, Halifax, B3H 4R2 Nova Scotia Canada; 3 Collaborative Innovation Center for Biomedicine, School of Clinical Medicine, Shanghai University of Medicine and Health Sciences, 279 Zhouzhu Rd, Shanghai, 201318 China

**Keywords:** Biophysics, Cell biology

## Abstract

Macrophages are highly specialized in removing large particles including dead cells and cellular debris. When stimulated, delivery of the intracellular lysosomal membranes is required for the formation of plasmalemmal pseudopods and phagosomes. As a key lysosomal Ca^2+^ channel, Transient Receptor Potential Mucolipin-1 (TRPML1) regulates lysosomal exocytosis and subsequent phagosome biogenesis, thereby promoting phagocytosis of large extracellular particles. Recently, we have suggested that TRPML1-mediated lysosomal exocytosis is essentially dependent on lysosomal big conductance Ca^2+^-activated potassium (BK) channel. Therefore, we predict that lysosomal BK channels regulate large particle phagocytosis. In this study, by using RAW264.7 macrophage cell line and bone marrow-derived macrophages, we show that although BK is dispensable for small particle uptake, loss of BK significantly inhibits the ingestion of large particles whereas activating BK increases the uptake of large particles. BK facilitating effect on large particle ingestion is inhibited by either blocking TRPML1 or suppressing lysosomal exocytosis. Additionally, the increased uptake of large particles by activating TRPML1 is eliminated by inhibiting BK. These data suggest that BK and TRPML1 are functionally coupled to regulate large particle phagocytosis through modulating lysosomal exocytosis.

## Introduction

Macrophages are highly phagocytic cells that originate in the bone marrow or derived from monocytes. They play an important role in the immune response to foreign invaders of the body, such as infectious microorganisms, or to accumulating damaged or apoptotic cells. Upon pathogen binding, a cascade of signaling events are triggered, leading to the extension of the plasma membrane (PM) surrounding the particle(s) to form phagocytic cups that ingest particles into vacuole-like structures called phagosomes. Phagosomes then undergo a maturation process by fusing with lysosomes to form phagolysosomes where the pathogen is killed by toxic peroxides and further digested by acidic hydrolytic enzymes^1,2^. Accumulating evidence suggests that intracellular membranes including lysosomes contribute to the cell surface at the sites of particle uptake and regulate phagosome formation. For example, fusion of lysosomes with the PM, so called lysosomal exocytosis, is essential for large particle uptake by macrophages^3–6^.

As with the synaptic vesicle fusion with the PM, lysosome fusion with the PM is a Ca^2+^-dependent process, and the release of intralysosomal Ca^2+^ (~0.5 mM) is important for lysosomal exocytosis^3,7,8^. The ubiquitously expressed TRPML1 acts as a lysosomal Ca^2+^ release channel that regulates lysosomal exocytosis^3,9–11^. Emerging evidence also suggests that the ubiquitously expressed synaptotagmin isoform VII (Syt VII) is enriched in lysosomes where it serves as the Ca^2+^ sensor to mediate lysosomal exocytosis^4,12,13^. In agreement with the notion that lysosomes provide membranes necessary for pseudopod extension and subsequent clearance of apoptotic cells, large particle ingestion is impaired in macrophages derived from either TRPML1^−/−^ mice^3^ or Syt VII^−/−^ mice^4^.

Because TRPML1 channels are strongly inwardly rectifying^14^, their activation causes a large amount of Ca^2+^ (and Na^+^) loss from lysosomal lumen, which could collapse the potential gradient across the lysosomal membrane^12,15,16^, preventing further Ca^2+^ (and Na^+^) release. Thus, either counter cation influx or anion co-release should exist to balance the loss of luminal cations resulting from continuous Ca^2+^ (and Na^+^) release. Interestingly, we recently report that BK channels are localized in lysosomes where they form a macromolecular complex with TRPML1 and regulate TRPML1-mediated Ca^2+^ release using a positive feedback mechanism, i.e. Ca^2+^ release via TRPML1 activates BK; activated BK in turn facilitates TRPML1-mediated lysosomal Ca^2+^ release and subsequent lysosomal exocytosis^12^. Thus, we predict that BK may regulate large particle uptake through regulating TRPML1-mediated lysosomal exocytosis. By using RAW264.7 macrophage cell line and bone marrow-derived macrophages (BMMs), in this study, we show that BK downregulation inhibits large particle uptake whereas BK upregulation increases the uptake of large particles. We also show that BK’s facilitation in large particle phagocytosis is dependent on TRPML1, Ca^2+^ and lysosomal exocytosis. In addition, the increased large particle uptake by activating TRPML1 is eliminated by inhibiting BK. Our results suggest that BK and TRPML1 coordinately regulate the clearance of apoptotic cells and large cellular debris.

## Results

### BK channels are required for efficient uptake of large particles in macrophages

**Figure 1:**
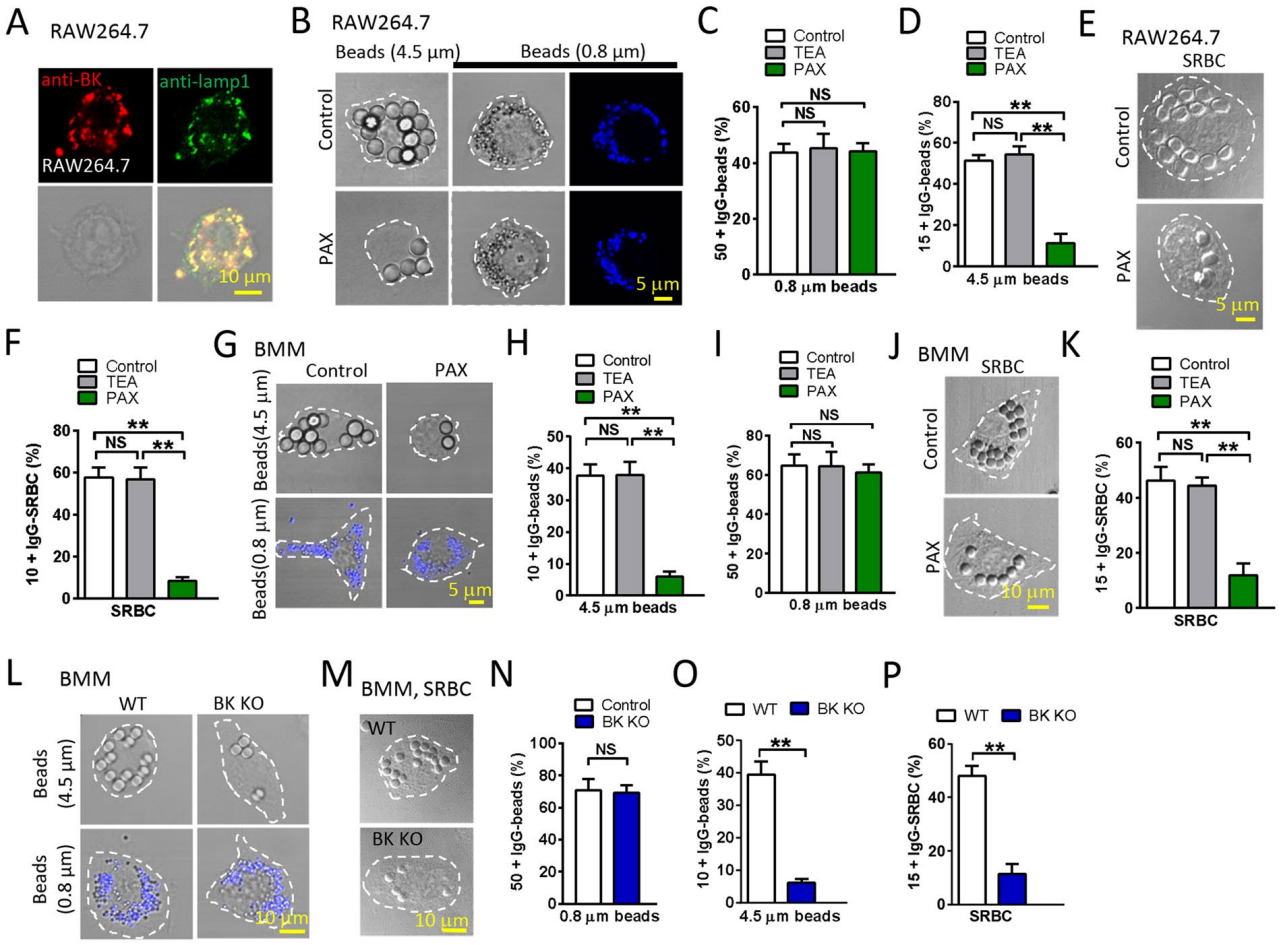
Inhibition of lysosomal BK channel significantly blocks macrophage phagocytosis of large particles. (**A**) Lysosomal localization of BK channels in RAW264.7 cells. RAW264.7 cells were co-stained with BK and Lamp1 antibodies. Scale bars: 10 μm. (**B**) Paxilline treatment dramatically inhibited the phagocytosis of large but not small beads in RAW264.7 cells. RAW264.7 cells were treated with 1 μM Paxilline for 30 min and then incubated with opsonized 4.5 μm (large) or 0.8 μm (small) polystyrene beads at 37 °C for 60 min with Paxilline. DMSO treated RAW264.7 cells were used as controls. Scale bars: 5 μm. (**C**,**D**) Summary of particle ingestion for the conditions indicated. Note that the membrane impermeable BK channel blocker TEA (5 mM) had no effect on particle uptake. (**E**,**F**) Paxilline reduced the uptake of SRBCs by RAW264.7 cells. RAW264.7 cells were treated with 1 μM Paxilline for 30 min and then incubated with SRBCs at 37 °C for 60 min. DMSO treated RAW264.7 cells were used as controls. Scale bars: 5 μm. (**G**–**I**) Paxilline treatment dramatically prevented BMMs from ingesting large beads, but not small beads. TEA had no effect on large particle uptake. Scale bars: 5 μm. (**J**,**K**) Paxilline but not TEA inhibited the uptake of SRBCs by BMMs. Scale bars: 10 μm. (**L**–**P**) In BMMs, loss of BK decreased the uptake of large beads and SRBCs but not small beads. Scale bars: 10 μm. For particle ingestion, experiments were repeated three times with triplicated samples each time for all conditions. Totally, 150–200 cells were counted for each condition.

As in other cells^12,15^, BK is highly enriched in lysosomes of macrophages (Fig. 1A). To probe the possibility of BK in large particle phagocytosis, we first exposed RAW264.7 cells to different-sized IgG-opsonized polystyrene beads for 60 min, and then compared the uptake of beads in cells with and without Paxilline (1 µM), the selective membrane permeable BK channel blocker. Based on distribution histograms of the number of ingested particles per cell (Fig. S1A), thresholds were set to compare the phagocytic capability according to the cell type and particle type, i.e. 15 or more particles per cell (15 + ) for 4.5 μm beads, 50 or more particles per cell (50 + ) for 0.8 μm beads, and 10 or more particles per cell (10 + ) for SRBCs. Interestingly, although the internalization of 0.8 μm beads (Fig. 1B,C, S2A) was comparable between control and Paxilline-treated macrophages, the uptake of 4.5 μm beads was significantly reduced by Paxilline compared with the control in a dose dependent manner (Fig. 1B,D, S2A, S2B).

To confirm the necessity of BK in large particle uptake, RAW264.7 cells were further exposed to IgG-opsonized sheep red blood cells (IgG-SRBCs) for 60 min. Un-ingested IgG-SRBCs were hypotonically lysed by briefly (1–2 min) incubating the cells in 4 °C water. We found that Paxilline significantly reduced the ingestion of SRBCs (Fig. 1E,F, S2C).

To further test the requirement of BK in large particle uptake, BMMs isolated from wild-type (WT) mice were exposed to IgG-opsonized polystyrene beads for 60 min as before^3^. Based on a threshold of 10 or more (10 + ) for 4.5 μm beads, a threshold of 15 or more (15 + ) for IgG-SRBCs, and a threshold of 50 or more (50 + ) for 0.8 μm beads (Fig. S1B), we found that Paxilline markedly reduced the ingestion of large beads (Fig. 1G,H, S2D) but not small beads (Fig. 1G,I, S2D) by BMMs. In agreement with this, inhibition of BK channels using Paxilline (1 μM) significantly reduced the number of IgG-SRBCs internalized by BMMs (Fig. 1J,K, S2E). These data are in a good agreement with the data from RAW264.7 cells (Fig. 1C,D,F). In contrast, the membrane impermeable K^+^ channel blocker tetraethylammonium (TEA, 5 mM)^12^, did not mimic the effect of Paxilline in both RAW264.7 (Fig. 1C,D,F) and BMMs (Fig. 1H,I,K), suggesting that lysosomal BK but not the PM BK channels are required for the uptake of large particles.

The uptake of IgG-SRBCs and beads was further compared between BMMs isolated from wild-type and BK knockout (BK KO) mice. As shown in Fig. 1L–P and S2F-G, significantly fewer large beads and SRBCs but not small beads were ingested by BK deficient BMMs compared with wild-type controls. Taken together, these results suggest that BK is required for efficient uptake of large but not small particles.

### BK upregulation increases large particle uptake in macrophages

**Figure 2:**
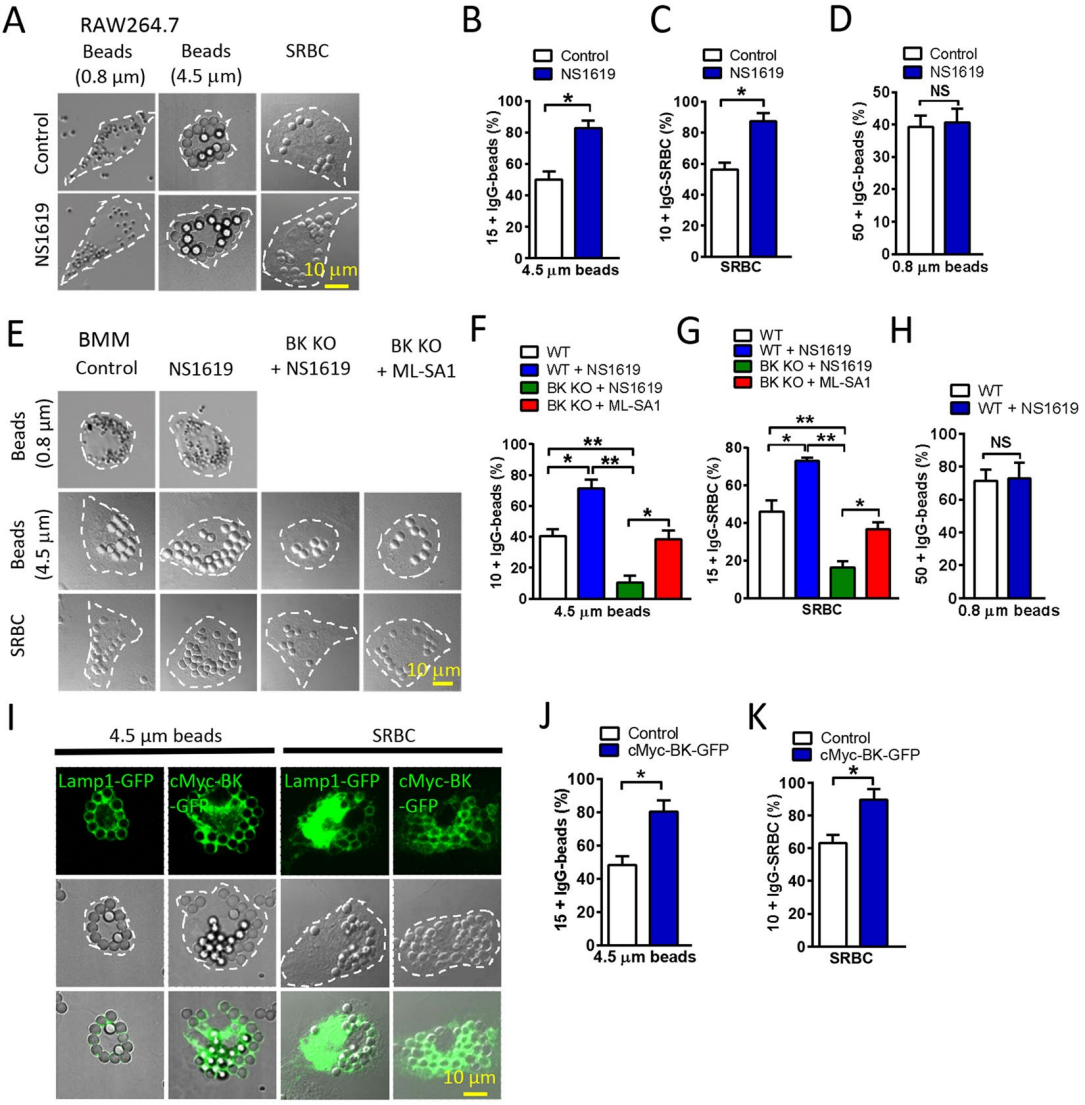
Activation or overexpression of BK increases macrophage phagocytosis of large particles. (**A**–**D**) BK agonist NS1619 significantly increased the ingestion of large beads and SRBCs but not small beads in RAW264.7 cells. RAW264.7 cells were treated with 20 μM NS1619 for 30 min and then incubated with opsonized 4.5 μm or 0.8 μm polystyrene beads or SRBCs at 37 °C for 60 min. Scale bars: 10 μm. (**E**–**H**) NS1619 significantly increased the ingestion of large beads (4.5 μm) and SRBCs in BMMs from wild-type but not BK KO mice. ML-SA1 rescued the defective phagocytosis of large particles in BMMs derived from BK KO mice. BMMs were pre-treated with 20 μM NS1619 or 10 μM ML-SA1 for 30 min and then incubated with large beads or SRBCs at 37 °C for 60 min. Scale bars: 10 μm. (**I**–**K**) Overexpression of BK in RAW264.7 cells increased the ingestion of large beads and SRBCs. For particle ingestion, experiments were repeated three times with triplicated samples each time for all conditions. Totally, 150–200 cells were counted for each condition.

We further examined whether BK upregulation facilitates particle uptake by macrophages. As shown in Fig. 2A–D and S3, NS1619, a BK agonist, significantly increased the ingestion of large beads and SRBCs by RAW264.7 cells, respectively, but not small beads. Consistently, incubating wild-type BMMs with NS1619 (20 μM) caused an increase in the uptake of large beads and SRBCs but not small beads (Fig. 2E–H, S4). In contrast, this increase was negligible in BK KO BMMs. Interestingly, ML-SA1 (10 μM) was able to rescue the defective ingestion of large particles in BK KO macrophages, suggesting that ML1 is the primary regulator to control large particle ingestion and BK may promote large particle uptake through facilitating TRPML1 function. This is in agreement with our previous studies showing that upregulating TRPML1 corrects lysosomal defects in BK deficient cells whereas BK upregulation fails to correct lysosomal defects in TRPML1 deficient cells^12^. In addition, heterologous expression of BK-GFP in RAW264.7 cells remarkably increased the uptake of both large beads and SRBCs (Fig. 2I–K). Collectively, these data suggest that the efficiency of particle uptake in macrophages is positively correlated with the expression level and channel activity of BK.

### BK channels regulating large particle uptake is not due to particle loading volume

**Figure 3:**
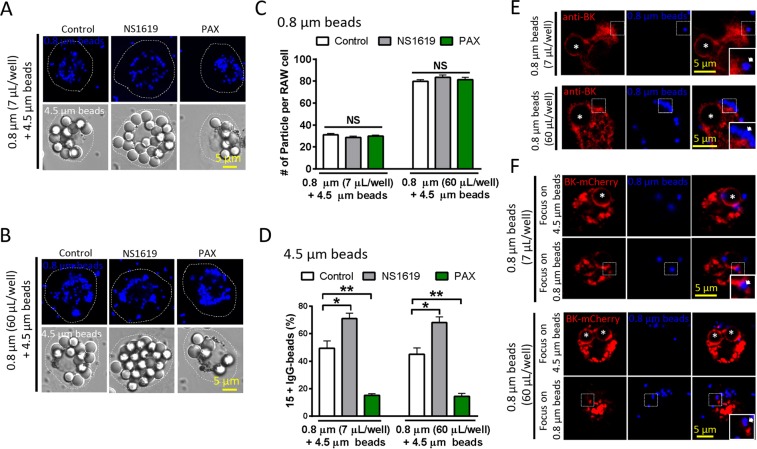
BK channels regulating large particle uptake is not due to particle loading volume. (**A**) Representative images of RAW264.7 cells co-loaded with 4.5 μm beads and moderate dose (7 μL/well) of 0.8 μm beads. (**B**) Representative images of RAW264.7 cells co-loaded with 4.5 μm beads and saturate dose (60 μL/well) of 0.8 μm beads. (**C**) Neither NS1619 nor PAX had effect on the uptake of 0.8 μm beads when RAW264.7 cells were co-loaded with 4.5 μm beads and moderate dose of 0.8 μm beads or saturate dose of 0.8 μm beads. (**D**) Both NS1619 and PAX had effect on 4.5 μm bead ingestion when RAW264.7 cells were co-loaded with moderate dose of 4.5 μm beads and moderate or saturate dose of 0.8 μm beads. Cells were pre-treated with NS1619 (20 μM) or PAX (1 μM) for 30 min. (**E**) Endogenous BK was only recruited to the surface of 4.5 μm beads (asterisks) but not 0.8 μm beads (arrows, at both moderate and saturate doses) after co-loading for 5 min in RAW264.7 cells. (**F**) Exogenous BK was recruited to the surface of large (4.5 μm) beads but not small (0.8 μm, at both moderate and saturate doses) beads. Asterisks indicate the center of the 4.5 μm beads, and arrows indicate the location of 0.8 μm beads. For particle ingestion, experiments were repeated three times with triplicated samples each time for all conditions. Totally, 150–200 cells were counted for each condition.

It is possible that the different role of BK in the uptake between small and large particles was attributed to their different loading volumes. To exclude this possibility, we performed concentration dependence of small beads and obtained a curve of small particle loading (Fig. S5A, B), and then we tested the effect of Paxilline and NS1619 on the ingestion of small beads at saturate concentration. We found that both Paxilline and NS1619 had no effect on the ingestion of small beads at the saturate concentration (Fig. S5C) while large particle uptake was regulated by both Paxilline and NS1619 (Fig. S5D, E). To further exclude the possibility, we employed co-loading of moderate concentration of large beads (bead: RAW264.7 = 25~50: 1) with both moderate (7 µL/well, Fig. 3A,C,D) and saturate concentration (60 µL/well, Fig. 3B–D) of small beads in RAW264.7 macrophages. We found that under both conditions, NS1619 and PAX did not affect the uptake of small beads (Fig. 3A–C, S5F, G) while the uptake of large beads was altered by both NS1619 and PAX (Fig. 3A,B,D, S5F, G). Therefore, BK specifically regulates the uptake of large but not small particles. These data also suggest that large and small particles occupy distinct pools of vesicle in the cells. In supporting this, BK decorated large but not small particles (both moderate and saturate concentrations) at 5 min after particle loading (Fig. 3E,F), although as in ML1, BK was recruited to small beads enclosed phagosomes after long term (45 min) particle loading (Fig. S5H).

### BK channels regulate large particle uptake via TRPML1

**Figure 4:**
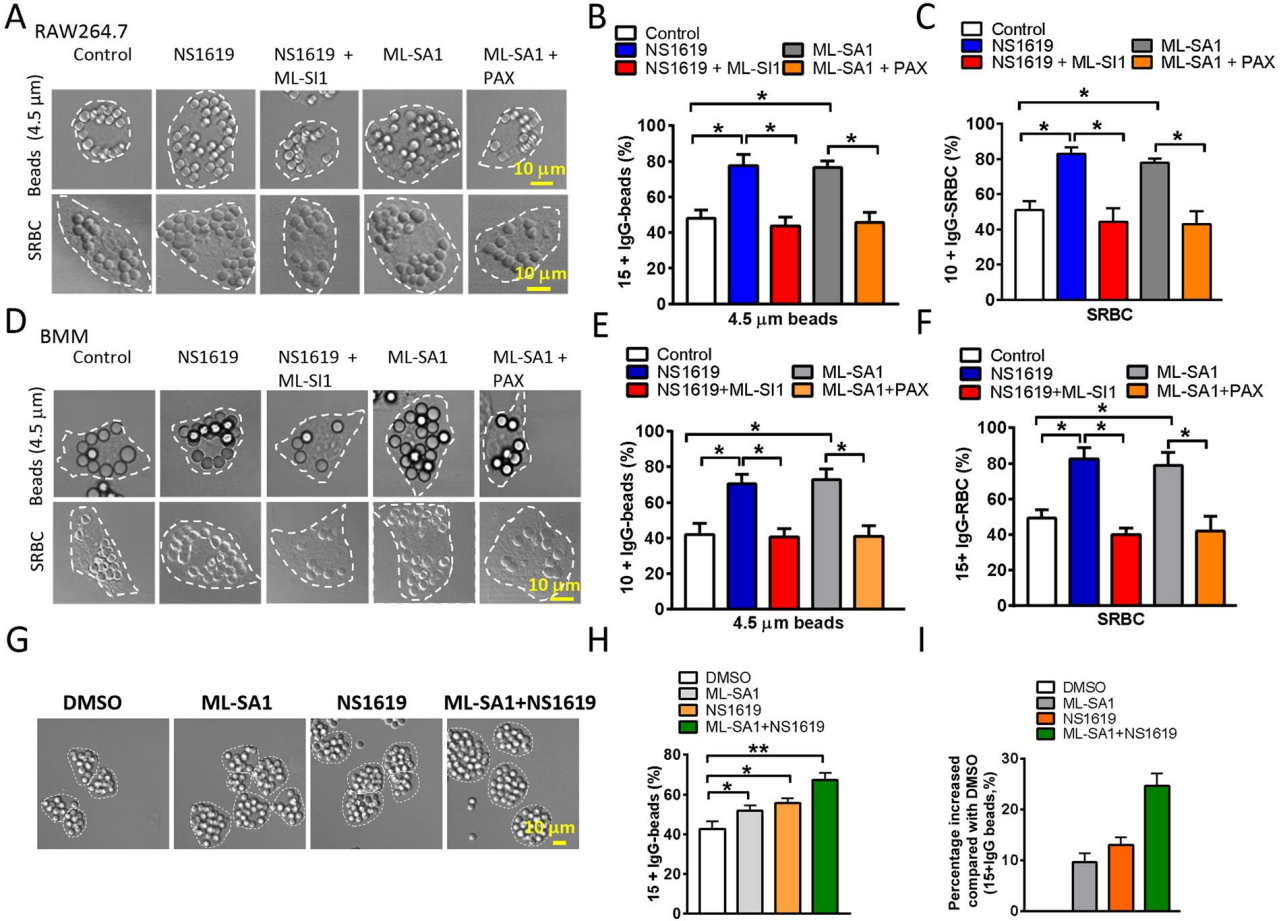
Enhanced phagocytosis by BK activation requires TRPML1 function. NS1619 (20 μM) and ML-SA1 (10 μM) treatment increased large particle ingestion in RAW264.7 cells (**A**–**C**) and BMMs (**D**–**F**). Co-applying TRPML1 inhibitor ML-SI1 (10 μM) abolished the effect of NS1619. Blocking BK by Paxiline (3 μM) treatment also inhibited ML-SA1’s effect. These data suggest that BK and TRPML1 are strongly coupled to regulate large particle ingestion. (**G**–**I**) Additive effect of NS1619 and ML-SA1 on the uptake of 4.5 μm beads in RAW264.7 cells. RAW264.7 cells were pre-treated with 5 μM NS1619 or 3 μM ML-SA1 or both for 30 min, and then incubated with opsonized 4.5 μm polystyrene beads at 37 °C for 60 min. Note that, after removing the DMSO background, the combination of ML-SA1 and NS1619 produced an effect similar to the sum of their individual effects (9.667 ± 1.667 for ML-SA1, 13 ± 1.528 for NS1619, and 24.67 ± 2.333 for ML-SA1 and NS1619 combined). For particle ingestion, experiments were repeated three times with triplicated samples each time for all conditions. Totally, 150–200 cells were counted for each condition.

Our previous study indicates that BK interacts with TRPML1 and promotes lysosomal exocytosis by increasing TRPML1-mediated lysosomal Ca^2+^ release^12^. To understand the molecular mechanism of BK in large particle uptake, we tested whether BK regulation of large particle ingestion is through modulating TRPML1. Indeed, NS1619-induced increase in the uptake of large beads or SBRCs was inhibited by ML-SI1 (10 μM, 30 min), the TRPML1 inhibitor^3^, in both RAW264.7 macrophages (Fig. 4A–C) and BMMs (Fig. 4D–F). In agreement with the notion that TRPML1-BK complex regulates large particle uptake, Paxilline blocked the increased uptake of large beads or SRBCs that was induced by activating TRPML1 using ML-SA1 (10 μM, 30 min) (Fig. 4A–F). Additionally, the total effect of ML-SA1 and NS1619 is similar to the sum of their individual effects (Fig. 4G–I), suggesting that the two drugs have no synergistic effect. Altogether, these data suggest that BK and TRPML1 are tightly associated to ensure efficient uptake of large particles in macrophages.

### BK regulates particle ingestion via lysosomal exocytosis

**Figure 5:**
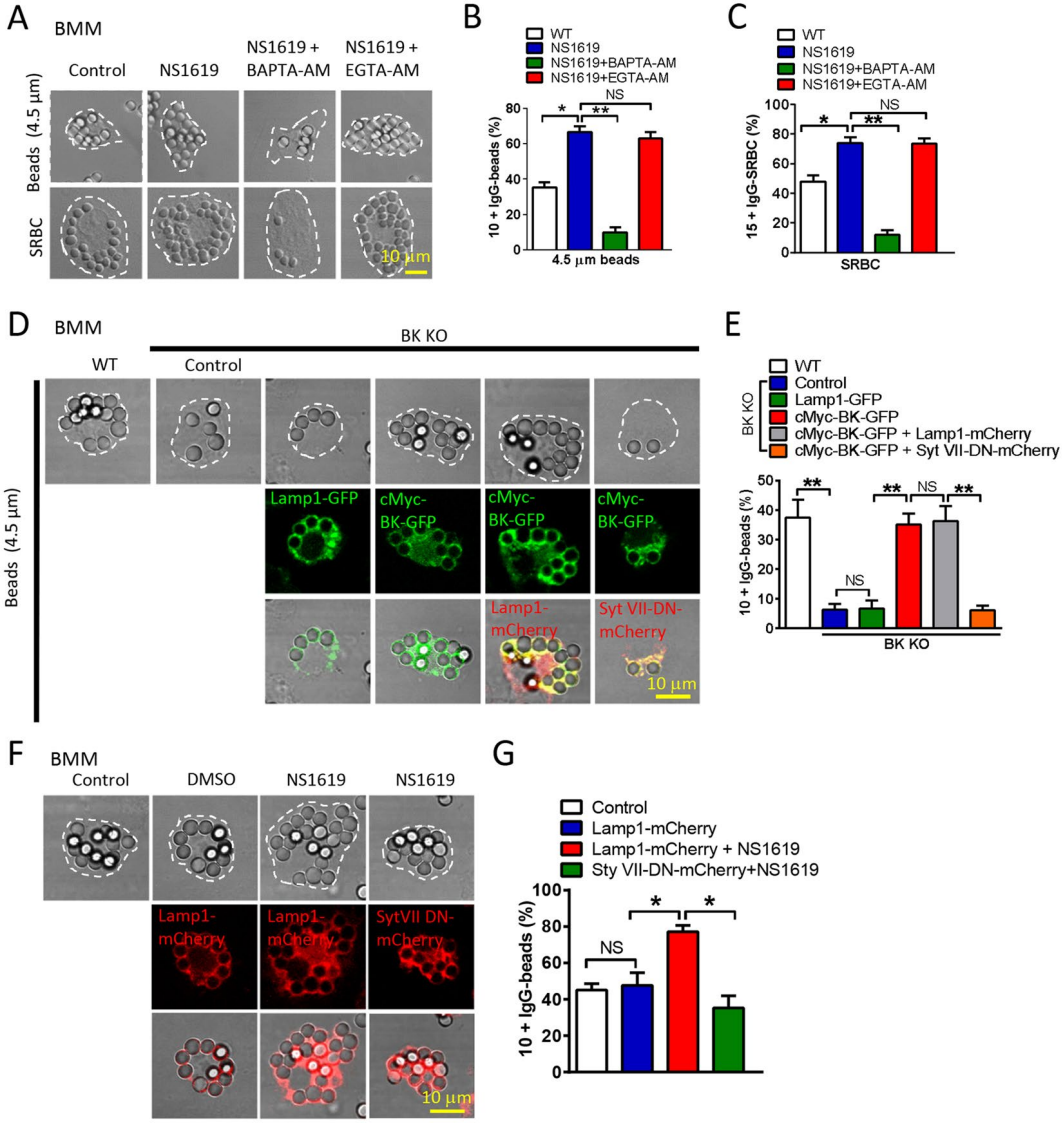
BK regulates particle ingestion via lysosomal exocytosis. (**A**–**C**) Intracellular calcium chelation abolishes the enhanced phagocytosis by BK activation. Fast intracellular Ca^2+^ chelator BAPTA-AM but not the slower chelator EGTA-AM blocked the upregulated large beads ingestion induced by NS1619 in BMMs. BMMs were treated with NS1619 (20 μM) with or without EGTA-AM (10 μM) or BAPTA-AM (1 μM) for 30 min prior to phagocytosis assays and then incubated with opsonized 4.5 μm or 0.8 μm polystyrene beads or SRBCs at 37 °C for 60 min. Scale bars: 10 μm. (**D**,**E**) Transfection of cMyc-BK-GFP but not Lamp1-GFP rescued the defective ingestion of 4.5 μm beads in BK KO BMMs. This was blocked by Syt VII-DN overexpression. BMMs were transfected with identical amounts of cMyc-BK-GFP, Lamp1-GFP, Lamp1-mCherry and Syt VII-DN-mCherry, respectively. Scale bars: 10 μm. (**F**,**G**) Syt VII-DN markedly reduced the effect of NS1619 on large particle uptake in BMMs. BMMs were transfected with Lamp1-mCherry or Syt VII-DN-mCherry for 24 hours, and then incubated with IgG-opsonized 4.5 μm beads for 60 mins. NS1619 (20 μM) were applied 30 min prior to phagocytosis assays. For particle ingestion, experiments were repeated three times with triplicated samples each time for all conditions. Totally, 150–200 cells were counted for each condition.

Lysosome fusion with the PM (lysosomal exocytosis) has been shown to be required for the uptake of large particles ( > 3 μm) in macrophages^3,4,17^. We have suggested that TRPML1 and BK are required for lysosomal exocytosis^12^. Next, we asked whether BK regulating large particle uptake is mediated by modulating lysosomal exocytosis. To test this, we assessed particle uptake upon upregulating BK while simultaneously blocking lysosomal exocytosis. First, the fast Ca^2+^ chelator BAPTA-AM was used to chelate Ca^2+^ released from lysosomes and subsequently inhibit Ca^2+^-dependent lysosomal exocytosis^18–26^. As shown in Fig. 5A–C, NS1619-induced large particle (beads or SRBCs) uptake was significantly reduced by the fast Ca^2+^ chelator BAPTA-AM (1 μM) but not the slower chelator EGTA-AM (10 μM) in BMMs^27^. This data suggests that NS1619 potentiating large particle ingestion is a very fast Ca^2+^-dependent process, likely through facilitating nearby TRPML1-mediated Ca^2+^ release from lysosomes^3,4,12^. Second, dominant-negative (DN) forms of Syt VII (Syt VII-DN) that was adopted to inhibit lysosomal exocytosis^12^ markedly reduced the effect of BK overexpression on large particle uptake in BK deficient BMMs (Fig. 5D,E). Consistently, the potentiation of NS1619 on large particle uptake was also blocked by Syt VII-DN expression (Fig. 5F,G). To support our hypothesis that BK promotes large particle uptake through facilitating lysosomal exocytosis, we directly monitored lysosomal exocytosis by measuring the level of lysosomal enzyme β-hexosaminidase in the culture medium. We found that large but not small particles increased lysosomal exocytosis, and this was inhibited by Paxilline but facilitated by NS1619. In addition, increased lysosomal exocytosis by NS1619 was eliminated by BAPTA-AM (Fig. S6A, B). Taken together, these data suggest that BK facilitates large particle uptake through promoting lysosomal exocytosis.

### BK is recruited to phagocytic cups and nascent phagosomes upon particle stimuli

**Figure 6:**
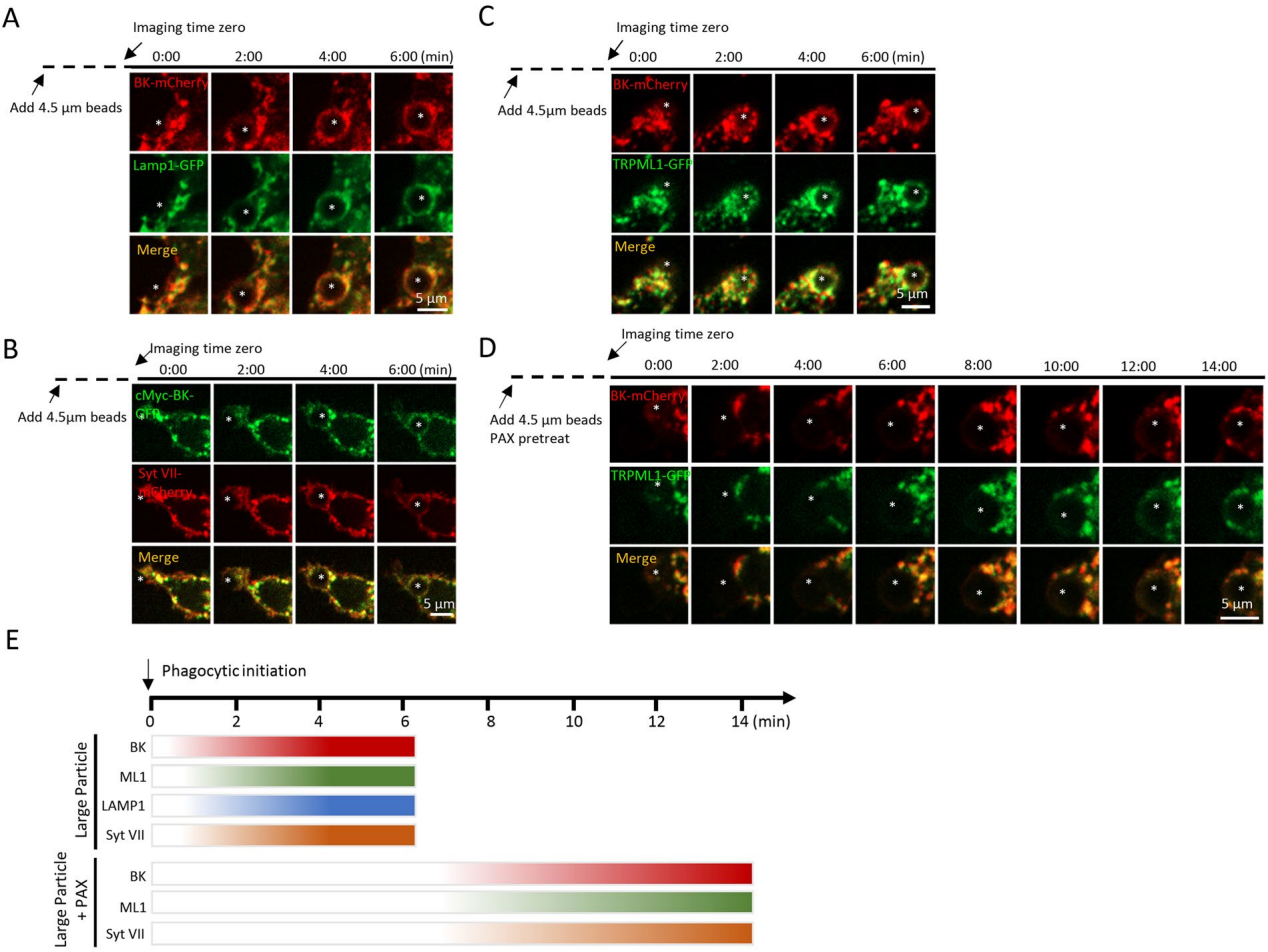
Large particle binding causes the recruitment of lysosomal membrane proteins to the phagocytic sites in RAW264.7 macrophages. (**A**–**D**) Selected frames from time-lapse confocal microscopy of RAW 264.7 macrophages transfected with indicated plasmids that were exposed to 4.5 µm IgG-coated polystyrene beads. In response to 4.5 μm IgG-coated particles, BK channels and other lysosomal membrane proteins were simultaneously recruited to the forming phagosomes within 4 min after phagocytosis initiation. The center of the particle is indicated with an asterisk. (**E**) Comparable recruitment kinetics of BK, TRPML1, Syt VII and Lamp1 to the particle-containing phagosomes in RAW264.7 macrophages were detected. Paxilline (1 µM) treatment inhibited the recruitment of lysosomal membrane proteins.

Previous studies suggested that in response to the binding of large particles, TRPML1 was recruited to phagocytic cups and nascent phagosomes^3^. If TRPML1-BK coupling is required for large particle ingestion, we expect to see the same kinetics of BK channel recruitment to the phagocytic cup and the phagosomal surface as TRPML1. To address this question, we performed time-lapse confocal microscopy of live RAW264.7 cells during the particle uptake process. We observed that BK was rapidly (within 4 min) recruited to the sites of phagosome formation together with Lamp1 (Fig. 6A), Syt VII-mCherry (Fig. 6B) and TRPML1-GFP (Fig. 6C) during uptake of 4.5 µm beads. Therefore, the kinetics of BK channel recruitment to the phagocytic cup and the phagosome following the recruitment kinetics of TRPML1, as well as other lysosomal membrane proteins. Interestingly, Paxilline reduced the speed of recruitment of the lysosomal membrane proteins to lysosomes (Fig. 6D,E).

**Figure 7:**
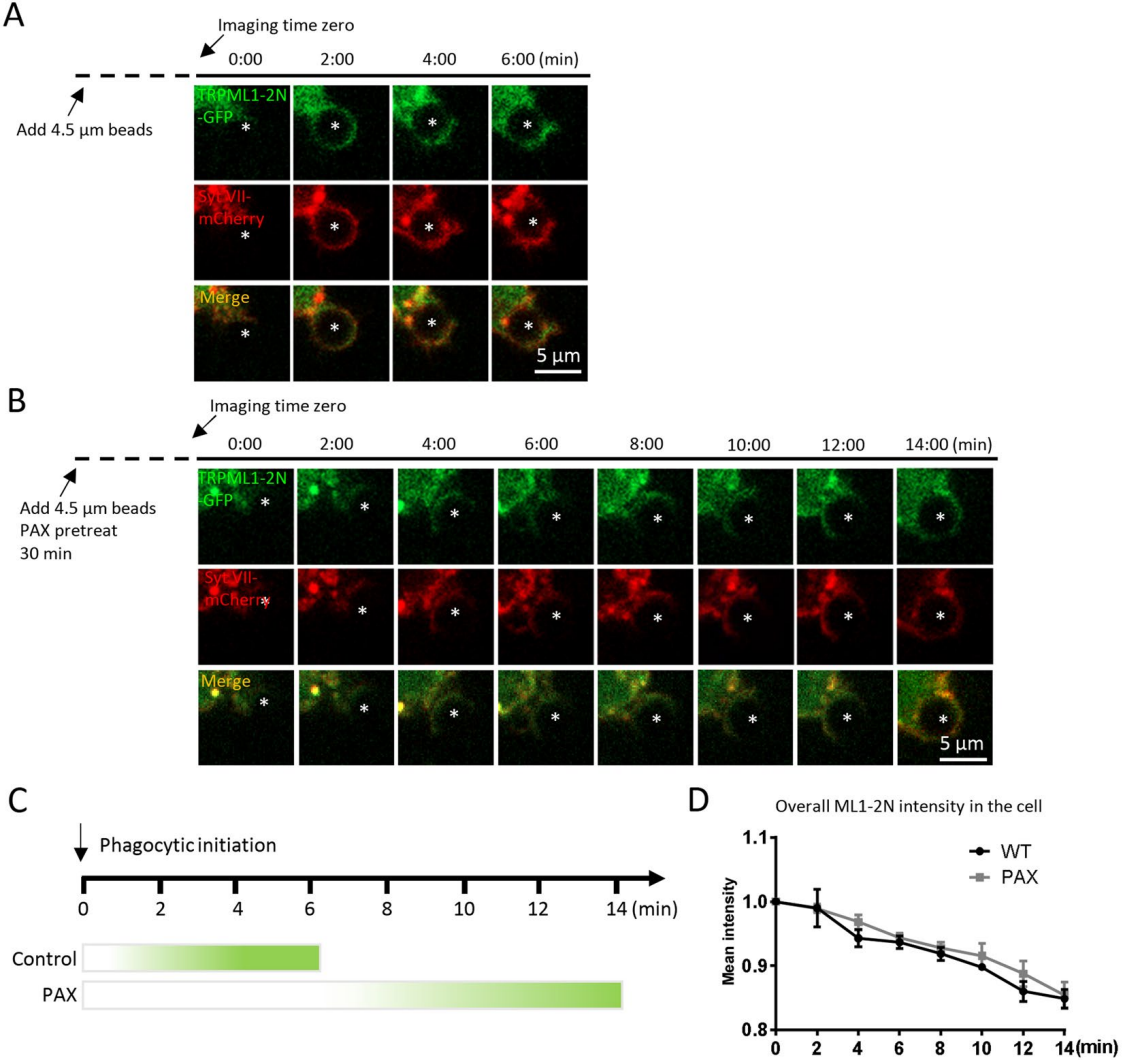
Inhibiting BK suppresses the recruitment of PI(3,5)P2 to forming phagosomes. (**A**,**B**) Selected frames from time-lapse confocal microscopy of RAW264.7 macrophages transfected with TRPML1-2N-GFP and Syt VII-mCherry that were exposed to 4.5 µm IgG-coated polystyrene beads. In response to 4.5 μm IgG-coated particles, TRPML1-2N-GFP and Syt VII-mCherry were simultaneously recruited to the forming phagosomes within 4 min after phagocytosis initiation. The center of the particle is indicated with an asterisk. (**C**) Paxilline (1 µM) treatment inhibited the recruitment of TRPML1-2N-GFP. (**D**) The overall TRPML1-2N-GFP intensity was not altered by Paxilline. These data suggest that BK regulates the recruitment of PI(3,5)P2 to forming phagosomes but not the overall PI(3,5)P2 production.

Because 1) large particle binding induces a local elevation of PI(3,5)P2, the endogenous activator of TRPML1, 2) because TRPML1 regulates particle ingestion depending on PI(3,5)P2^3,28^, and 3) enhance of phagocytosis by BK activity depends on TRPML1 activity, we suspected that BK might regulate the recruitment of PI(3,5)P2 on the surface of the phagocytic cups and nascent phagosomes during particle uptake. In agreement with previous report^3^, we found that TRPML1-2N-GFP, the PI(3,5)P2-specific probe^3,29^, was rapidly recruited to the forming phagosomes (Fig. 7A) in RAW264.7 cells that were transfected with TRPML1-2N-GFP and Syt VII-mCherry. A transient and localized increase in TRPML1-2N-GFP fluorescent intensity was observed within 4 min of particle binding. Interestingly, in the presence of Paxilline, the localized TRPML1-2N-GFP fluorescence surrounding beads was dramatically slowed down (Fig. 7A–C). To examine whether BK regulates PI(3,5)P2 production, we quantitate the overall intensity of TRPML1-2N-GFP in the cell. We found that Paxilline did not alter the overall TRPML1-2N-GFP intensity (Fig. 7D). Altogether, these data suggest that BK may regulate large particle uptake by compromising PI(3,5)P2 recruitment/production in the lysosome but not the overall production of PI(3,5)P2 in the cell. Taken together, by coupling with TRPML1^12^, BK promotes TRPML1-dependent lysosomal exocytosis to help large particle phagocytosis.

## Discussion

Previous studies have suggested that lysosomal Ca^2+^ channel TRPML1 promotes large but not small particle uptake by increasing lysosomal exocytosis^3,30^. New evidence also suggests that lysosomal BK channel forms a positive feedback regulatory loop with TRPML1 to facilitate lysosomal Ca^2+^ release and exocytosis. In this study, we further demonstrate that lysosomal BK facilitates large but not small particle uptake that requires TRPML1 and lysosomal exocytosis. In the meantime, we show that TRPML1 promoting large particle phagocytosis is also dependent on BK channel. Therefore, this study consolidates the notion that lysosomal BK channel and TRPML1 channel form a functional feedback loop in regulating lysosomal functions^12^ including large particle phagocytosis. This study also consolidates the notion that the lysosome membrane represents an important source for pseudopod extension and phagosome formation in response to large particles.

Together with previous studies^3,30^, during early stage of phagocytosis large and small particles may occupy distinct pools of vesicle in the cells and the lysosome is only involved in large particle internalization. This has been supported by three pieces of evidence. First, in macrophages co-loaded with large beads and small beads, BK decorates only large but not small particles at 5 min after particle loading (Fig. 3E,F); second, TRPML1^3,30^ and BK specifically regulates the uptake of large but not small particles (Figs. 1, 2 and 3, S5); third, large but not small beads induce lysosomal exocytosis (Fig. S6).

Interestingly, upregulating TRPML1 by ML-SA1 rescued the reduced large particle ingestion in BK KO (Fig. 2E–G) and Paxilline-treated (Fig. 4A–F) cells, one would suggest that BK is not really important for TRPML1 dependent phagocytosis of large particles. However, ML-SA1 increases the ingestion of large particles to a much higher level in WT cells^3^, compared with BK KO and Paxilline-treated cells. Therefore, we suggest that BK can promote TRPML1 dependent phagocytosis of large particles. Taken together, we believe that in the TRPML1-BK coupling machinery TRPML1 plays the primary role in the ingestion of large particles, whereas BK provides the secondary control over large particle uptake through facilitating TRPML1 function as we showed before^12^. However, given that ML-SA1 completely corrects the impaired large particle ingestion in BK KO cells, some other lysosomal K^+^ channels may also play a role in large particle uptake.

By using GCaMP3-TRPML1, Samie *et al*.^3^ showed that large particle binding caused transient and localized Ca^2+^ increases preferentially at the uptake sites of Syt VII-mCherry-positive lysosomes within several minutes. Unfortunately, the lack of such a K^+^ probe prevented us from monitoring localized BK activity. However, because of the tight coupling between TRPML1-BK^12^, and because of the same kinetics for their delivery to forming phagosomes (Fig. 7C), we believe that BK is likely activated upon large particle binding, and this may facilitate TRPML1-mediated lysosomal Ca^2+^ release^12^ and lysosomal exocytosis, and subsequent large particle ingestion. Indeed, the early recruitment of Syt VII within several minutes of phagocytosis initiation is impaired by both TRPML1 inhibition^3^ and BK inhibition (Fig. 6D,E, 7A,B). In addition to phagosome formation, BK may be also important for phagosome maturation and degradation because TRPML1 promotes phagosome-lysosome fusion^17,31^ and the degradation of phagocytic materials^30,32,33^, and because BK is recruited to small bead enclosed phagosomes after long term (45 min) particle loading (Fig. S5H). Therefore, it is likely that the TRPML1-BK coupling plays dual roles sequentially in phagosome formation and phagosome maturation^1,3,4^*.*

Intriguingly, BAPTA-AM but not ML-SI1, Paxilline and Syt VII-DN inhibited the ingestion of large particles to below the basal levels. This discrepancy between the effects of BAPTA-AM and the lysosomal modulators may be explained by the dependence of the late stages of the lysosomal exocytosis on Ca^2+^ influx through the PM^34^. Alternatively, BAPTA-AM may affect not only Ca^2+^-dependent lysosomal exocytosis but also other Ca^2+^-dependent cellular processes that are important for large particle uptake^35–38^.

Previous studies suggest that TRPML1 KO mice exhibited enlarged spleens, accumulation of RBCs in the spleen, and activation of microglia, compared with WT controls^3^. Because BK regulates the uptake of large particles by coupling with TRPML1, we predict that BK KO mice exhibit similar phenotypes as TRPML1 KO mice. To test this hypothesis, the spleen and the brain were collected from BK KO mice at the age used for investigating TRPML1 KO mouse phenotypes^3^. Our preliminary data showed that the spleen size and the level of RBCs in the spleen were comparable between WT and BK KO mice at 2-months. However, the expression of Iba1, a 17 kDa EF hand protein that is specifically expressed in activated macrophages/microglia, was increased in brain tissues from 4-month-old BK KO mice (data not shown). Currently, the reason causing the discrepancy between different tissues remains unclear. We suspect that, in tissues that are more vulnerable to environment changes such as the brain, BK may be required for the clearance of senescent and apoptotic cells. However, the discrepancy could also be due to the age of the mice used, i.e. BK KO mice may show the spleen phenotypes when they get older. In addition, some other K^+^ channels in lysosomes may compensate for the loss of BK channel in the spleen. These await further investigation.

Early studies suggested that BK channel is essential for antibacterial activity in neutrophils and eosinophils^39^. However, later studies indicated that BK channels are dispensable for innate immune functions of neutrophils, eosinophils and macrophages^40–42^. Although we did not observe that BK regulates small (0.8 μm) particle ingestion at early phagocytosis stage, BK was recruited to small beads enclosed phagosomes after long term (45 min) particle loading. Therefore, whether BK is involved in bacterial clearance in macrophages remains to be determined because BK has been suggested to regulate a complex signal transduction process elicited by bacterial infection in macrophages^40,43–45^, and because TRPML1 displays antimicrobial activity in macrophages^17^. Nevertheless, our results suggest that lysosomal BK channel is important for large particle phagocytosis through coupling with TRPML1. Because accumulation of large particles such as apoptotic cells may cause brain inflammation and microglia activation in most neurodegenerative lysosome storage diseases (LSDs)^46^, activating BK channel could be a tool to correct this defect in many LSDs^12^. Additionally, BK channel may play an important role in the phagocytosis of large bacteria and fungi^44,45^ and in normal development, neurodegenerative diseases and cancer that involve apoptosis^47,48^.

## Experimental Procedures

### Cell culture

RAW264.7 cells were obtained from ATCC (Manassas, VA) and maintained in Dulbecco’s Modified Eagle’s Medium (DMEM) supplemented with 10% fetal bovine serum (FBS, Invitrogen, Carlsbad, CA, USA) at 37 °C and 5% CO_2_.

BMMs were prepared and cultured as described previously^3^. Briefly, femur and tibia were harvested from mice and rinsed in 70% ethanol. The bones were washed with sterilized PBS and then bone marrow cells were flushed out. The bone marrow cells were pipetted up and down to bring the cells into single-cell suspension. Erythrocytes were removed by ammonium-chloride-potassium (ACK) buffer treatment; the cell suspension was filtered through 70 μm cell strainer to remove any cell clumps. The cells were then cultured in complete DMEM (DMEM containing 100 U/mL penicillin, 100 mg/mL streptomycin, 2 mM of L-glutamine, sodium pyruvate and 10% FBS) with recombinant colony stimulating factor (BioLegend, San Diego, USA) at 37 °C and 5% CO_2_.

BMMs and RAW264.7 macrophages were transiently transfected with indicated DNA by Neon electroporation (Invitrogen Neon Transfection System, Invitrogen, USA) according to the manufacture procedures and cultured for 24 hrs before use.

### Mouse line

The generation and characterization of BK KO mice have been described previously^49^. Animals were used under approved animal protocols and Institutional Animal Care Guidelines at Dalhousie University.

### Antibodies and reagents

The following primary antibodies were used for immunofluorescence staining: anti-Slo1 (UC Davis/NIH NeuroMab Facility, Davis, CA or Abcam, Cambridge, UK), anti-Lamp1 (H4A3, 1D4B, Developmental Studies Hybridoma Bank, USA), Alexa 488 goat anti-rat antibody, Alexa 488 goat anti-rabbit antibody, Alexa 546 goat anti-mouse antibody and Texas Red goat anti-mouse antibodies were purchased from Invitrogen (Invitrogen, USA). The following chemicals were also used: Paxilline (Cayman Chemical Company, USA); TEA (Sigma, USA); ML-SA1 (Tocris Bioscience, UK); ML-SI1 (Enzo Life Sciences Inc, USA); NS1619 (Sigma, USA); BAPTA-AM (Tocris Bioscience, UK); EGTA-AM (Invitrogen, USA); Recombinant Mouse M-CSF (carrier-free) (BioLegend, USA); 4-Methylumbelliferyl *N*-acetyl-β-d-glucosaminide (Sigma, USA).

### Molecular biology

Plasmids c-Myc-BK-GFP, BK-mCherry, LAMP1-GFP and TRPML1-GFP constructs were prepared as before^12^. Syt VII DN-mCherry was made by amplifying Syt VII-DN cDNA from dominant negative pShooter-Flag-Syt VII-D172 N/D303N-GFP that was generously provided by Mitsunori Fukuda, and then the Syt VII-DN cDNA was inserted between NheI and HindIII site of mCherry-N1 vector. Lamp1-mCherry was a gift from Michael X. Zhu. TRPML1-2N-GFP was a gift from Haoxing Xu.

### Phagocytosis assay

SRBCs (Lampire Biological Laboratories, USA) were washed twice with PBS and then opsonized with sheep anti rabbit IgG antibody (Equitech-Bio Inc, USA) at 37 °C for 1 hr. Phagocytosis was initiated by adding IgG-SRBCs onto adherent BMMs and RAW264.7 macrophages at a ratio of 25~50:1 (RBC: BMMs or RAW264.7 macrophages). To synchronize binding and internalization, IgG-SRBCs were centrifuged at 300 rpm for 3 min together with adherent BMMs and RAW264.7 macrophages. Then macrophages were placed at 37 °C and 5% CO^2^ for 1 h. Non-ingested IgG-SRBCs were lysed by incubating the cells with water (1 mL) for 2–3 min at 4 °C. SRBC-containing BMMs and RAW264.7 macrophages were fixed in ice-cold methanol and bright-field microscopy images were taken.

Polystyrene beads (diameter, 4.5 μm; Polysciences, USA) were coated for 1 h at 37 °C with mouse IgG. Phagocytosis was initiated by adding beads onto adherent BMMs and RAW264.7 macrophages at a ratio of 25~50:1 (beads: BMMs or RAW264.7 macrophages). After centrifugation at 300 rpm for 3 min. BMMs and RAW264.7 macrophages were placed at 37 °C and 5% CO_2_ for 1 h. Nonadherent beads were removed with cold PBS and then cells were fixed in 4% Paraformaldehyde (PFA).

Approximately 150–200 BMMs and RAW264.7 macrophages were typically analyzed for each experiment. Ingested particles were counted by experimenters who were blinded to the genotype and treatment. Thresholds were set according to previous reports to compare the phagocytic ability of RAW264.7 cells or BMMs^3^. To test the effect of drugs on particle ingestion, BMMs and RAW264.7 were normally pretreated with the drug for 30 min and the treatments continued while assessing phagocytosis.

### Immunofluorescence staining

Cells grown on coverslips were fixed in ice-cold methanol for 5 min and permeabilized in PBS with 0.1% Triton X-100 for 5 min. After 60 min treatment with 3% bovine serum albumin (BSA) in PBS at room temperature, cells were incubated with primary antibodies at 4 °C overnight. Following 3 PBS washes, cells were incubated with fluorescence-conjugated secondary antibodies for 45 min at room temperature in the dark, and then washed 3 times with PBS. Coverslips were mounted onto glass slides with 75% glycerol. Images were captured using confocal microscopy (LSM510, Zeiss; USA).

### β-Hexosaminidase assay

RAW264.7 cells were cultured in 24-well plates. To measure β-hexosaminidase activity, 100 μL culture medium was incubated with 100 μL of 1 mM 4-Methylumbelliferyl *N*-acetyl-β-d-glucosaminide (Sigma, USA) in 0.2 M citrate buffer (0.1 M citric acid, 0.1 M sodium citrate, pH 4.5) for 1 h at 37 °C. Reactions were quenched by addition 200 μL of 0.2 M Tris base. Cells pellets were lysed with 1% Triton X-100, and then treated as above. β-hexosaminidase activity was indicated by the fluorescence of the product that was measured by SpectraMax M-3 Multi-Mode Microplate Readers (Molecular Devices, USA) with an excitation wavelength of 365 nm and an emission wavelength of 460 nm. The release percentage of β-hexosaminidase was calculated as follows: [β-hexosaminidase in culture medium / (β-hexosaminidase in culture medium + total β-hexosaminidase in pellet)] ×100%.

### Confocal microscopy

Confocal experiments were performed as previously described^12^. Images were acquired using Zeiss LSM510 META confocal microscope with a 63X oil-immersion objective lens. Sequential excitation wavelengths at 488 nm, 543 nm and 633 nm were provided by argon and helium-neon gas lasers, respectively. Emission filters BP500-550 and BP 565_615 and LP650 were used for collecting green, red and far red images in channels one, two and three respectively. Images were captured using ZEN2009 software (Zeiss, USA).

### Time-lapse imaging

Live imaging of 4.5 µm bead uptake by RAW264.7 cells was performed on a heated stage using a spinning disk confocal imaging system, which consists of a Zeiss spinning disk Axio Observer Z1 confocal microscope, a Plan APOCHROMAT 63X Oil immersion NA 1.4, and an Axiocam MRm camera. RAW264.7 cells transfected with indicated plasmids were put on the heated stage, and then IgG-opsonized 4.5 μm beads were added to the cells before a series of images were taken.

### Data analysis

Data were presented as mean ± SEM. Statistical comparisons were made using analysis of variance (ANOVA) and Student’s t test. P values of < 0.05 were considered statistically significant. NS: not significant, *P < 0.05; **P < 0.01.

## Supplementary information

Supplementary Information.

## Data Availability

All data generated or analysed during this study are included in this published article (and its Supplementary Information files).

## References

[1] Flannagan RS, Jaumouille V, Grinstein S (2012). The cell biology of phagocytosis. Annual review of pathology.

[2] Niedergang F, Chavrier P (2004). Signaling and membrane dynamics during phagocytosis: many roads lead to the phagos(R)ome. Current opinion in cell biology.

[3] Samie M (2013). A TRP channel in the lysosome regulates large particle phagocytosis via focal exocytosis. Developmental cell.

[4] Czibener C (2006). Ca2+ and synaptotagmin VII-dependent delivery of lysosomal membrane to nascent phagosomes. The Journal of cell biology.

[5] Braun V, Niedergang F (2006). Linking exocytosis and endocytosis during phagocytosis. Biology of the cell / under the auspices of the European Cell Biology Organization.

[6] Huynh KK, Kay JG, Stow JL, Grinstein S (2007). Fusion, fission, and secretion during phagocytosis. Physiology.

[7] Reddy A, Caler EV, Andrews NW (2001). Plasma membrane repair is mediated by Ca(2+)-regulated exocytosis of lysosomes. Cell.

[8] Rodriguez A, Webster P, Ortego J, Andrews NW (1997). Lysosomes behave as Ca2+-regulated exocytic vesicles in fibroblasts and epithelial cells. The Journal of cell biology.

[9] Medina DL (2011). Transcriptional activation of lysosomal exocytosis promotes cellular clearance. Developmental cell.

[10] LaPlante JM (2006). Lysosomal exocytosis is impaired in mucolipidosis type IV. Molecular genetics and metabolism.

[11] Dong XP (2009). Activating mutations of the TRPML1 channel revealed by proline-scanning mutagenesis. The Journal of biological chemistry.

[12] Cao Q (2015). BK Channels Alleviate Lysosomal Storage Diseases by Providing Positive Feedback Regulation of Lysosomal Ca2+ Release. Developmental cell.

[13] Martinez I (2000). Synaptotagmin VII regulates Ca(2+)-dependent exocytosis of lysosomes in fibroblasts. The Journal of cell biology.

[14] Dong XP (2008). The type IV mucolipidosis-associated protein TRPML1 is an endolysosomal iron release channel. Nature.

[15] Wang W (2017). A voltage-dependent K+ channel in the lysosome is required for refilling lysosomal Ca2+ stores. The Journal of cell biology.

[16] Cang C (2013). mTOR regulates lysosomal ATP-sensitive two-pore Na(+) channels to adapt to metabolic state. Cell.

[17] Dayam RM, Saric A, Shilliday RE, Botelho RJ (2015). The Phosphoinositide-Gated Lysosomal Ca(2+) Channel, TRPML1, Is Required for Phagosome Maturation. Traffic.

[18] Luzio JP, Bright NA, Pryor PR (2007). The role of calcium and other ions in sorting and delivery in the late endocytic pathway. Biochemical Society transactions.

[19] Hay JC (2007). Calcium: a fundamental regulator of intracellular membrane fusion?. EMBO reports.

[20] Piper RC, Luzio JP (2004). CUPpling calcium to lysosomal biogenesis. Trends in cell biology.

[21] Peters C, Mayer A (1998). Ca2+/calmodulin signals the completion of docking and triggers a late step of vacuole fusion. Nature.

[22] Pryor PR, Mullock BM, Bright NA, Gray SR, Luzio JP (2000). The role of intraorganellar Ca(2+) in late endosome-lysosome heterotypic fusion and in the reformation of lysosomes from hybrid organelles. The Journal of cell biology.

[23] Morgan AJ, Platt FM, Lloyd-Evans E, Galione A (2011). Molecular mechanisms of endolysosomal Ca2+ signalling in health and disease. The Biochemical journal.

[24] Lloyd-Evans E, Platt FM (2011). Lysosomal Ca(2+) homeostasis: role in pathogenesis of lysosomal storage diseases. Cell calcium.

[25] Cheng X, Shen D, Samie M, Xu H (2010). Mucolipins: Intracellular TRPML1-3 channels. FEBS letters.

[26] Pittman JK (2011). Vacuolar Ca(2+) uptake. Cell calcium.

[27] Ermolyuk YS (2013). Differential triggering of spontaneous glutamate release by P/Q-, N- and R-type Ca2+ channels. Nature neuroscience.

[28] Dong XP (2010). PI(3,5)P(2) controls membrane trafficking by direct activation of mucolipin Ca(2+) release channels in the endolysosome. Nature communications.

[29] Li X (2013). Genetically encoded fluorescent probe to visualize intracellular phosphatidylinositol 3,5-bisphosphate localization and dynamics. Proceedings of the National Academy of Sciences of the United States of America.

[30] Venkatachalam K (2008). Motor deficit in a Drosophila model of mucolipidosis type IV due to defective clearance of apoptotic cells. Cell.

[31] Wong CO (2017). Lysosomal Degradation Is Required for Sustained Phagocytosis of Bacteria by Macrophages. Cell host & microbe.

[32] Sun T, Wang X, Lu Q, Ren H, Zhang H (2011). CUP-5, the C. elegans ortholog of the mammalian lysosomal channel protein MLN1/TRPML1, is required for proteolytic degradation in autolysosomes. Autophagy.

[33] Isobe Y (2019). PIKfyve accelerates phagosome acidification through activation of TRPML1 while arrests aberrant vacuolation independent of the Ca2+ channel. Journal of biochemistry.

[34] Ravi S, Pena KA, Chu CT, Kiselyov K (2016). Biphasic regulation of lysosomal exocytosis by oxidative stress. Cell calcium.

[35] Gagnon, E. et al. Endoplasmic reticulum-mediated phagocytosis is a mechanism of entry into macrophages. *Cell* **110**, 119-131, doi:S0092867402007973 [pii]10.1016/s0092-8674(02)00797-3 (2002). 10.1016/s0092-8674(02)00797-3 12151002

[36] Melendez Alirio J., Tay Hwee Kee (2008). Phagocytosis: a repertoire of receptors and Ca2+ as a key second messenger. Bioscience Reports.

[37] Vashi Nimi, Andrabi Syed Bilal Ahmad, Ghanwat Swapnil, Suar Mrutyunjay, Kumar Dhiraj (2017). Ca2+-dependent Focal Exocytosis of Golgi-derived Vesicles Helps Phagocytic Uptake in Macrophages. Journal of Biological Chemistry.

[38] Nunes Paula, Demaurex Nicolas (2010). The role of calcium signaling in phagocytosis. Journal of Leukocyte Biology.

[39] Ahluwalia J (2004). The large-conductance Ca2+-activated K+ channel is essential for innate immunity. Nature.

[40] Papavlassopoulos M (2006). MaxiK blockade selectively inhibits the lipopolysaccharide-induced I kappa B-alpha /NF-kappa B signaling pathway in macrophages. Journal of immunology.

[41] Essin K (2007). Large-conductance calcium-activated potassium channel activity is absent in human and mouse neutrophils and is not required for innate immunity. American journal of physiology. Cell physiology.

[42] Femling JK (2006). The antibacterial activity of human neutrophils and eosinophils requires proton channels but not BK channels. The Journal of general physiology.

[43] Paul D (2013). Phagocytosis dynamics depends on target shape. Biophysical journal.

[44] Champion JA, Walker A, Mitragotri S (2008). Role of particle size in phagocytosis of polymeric microspheres. Pharmaceutical research.

[45] Schlam D (2015). Phosphoinositide 3-kinase enables phagocytosis of large particles by terminating actin assembly through Rac/Cdc42 GTPase-activating proteins. Nature communications.

[46] Vitner EB, Platt FM, Futerman AH (2010). Common and uncommon pathogenic cascades in lysosomal storage diseases. The Journal of biological chemistry.

[47] Savitz S (1998). Role of apoptosis in health and disease. Jama.

[48] Sinkovics JG, Horvath JC (1998). Role of apoptosis in health and disease. Jama.

[49] Meredith AL, Thorneloe KS, Werner ME, Nelson MT, Aldrich RW (2004). Overactive bladder and incontinence in the absence of the BK large conductance Ca2+-activated K+ channel. The Journal of biological chemistry.

