# Supplementary material for: A lysosomal K+ channel regulates large particle phagocytosis by facilitating lysosome Ca2+ release
Source: Sci Rep. 2020 Jan 23;10:1038. doi: 10.1038/s41598-020-57874-2 (PMC6978423; doi:10.1038/s41598-020-57874-2)
Supplement: Supplementary file 1 — Supplementary Information. [file 41598_2020_57874_MOESM1_ESM.pdf]

A lysosomal K^+ channel regulates large particle phagocytosis by facilitating lysosome Ca^{2+} release

Xue Sun[1,2], Mengnan Xu[2], Qi Cao[2], Peng Huang[3,*], Xiaojuan Zhu[1,*], and Xian-Ping Dong[2*]

[1]Key Laboratory of Molecular Epigenetics of Ministry of Education, Institute of Cytology and Genetics, Northeast Normal University, Changchun, Jilin, China

[2]Department of Physiology and Biophysics, Dalhousie University, Sir Charles Tupper Medical Building, 5850 College Street, Halifax, B3H 4R2, Nova Scotia, Canada

[3]Collaborative Innovation Center for Biomedicine, School of Clinical Medicine, Shanghai University of Medicine and Health Sciences, 279 Zhouzhu Rd, Shanghai 201318, China.

Running title: Lysosomal BK channels in large particle phagocytosis

Key words: BK channel; Slo1; lysosome Ca^{2+}; TRPML1 channel; phagocytosis

*To whom correspondence should be addressed: xpdong@dal.ca, zhuxj720@nenu.edu.cn or huangp320@sina.com

Supplementary Figure legends

Figure S1. Histograms represent the distribution of macrophages containing different numbers of ingested large particles for each time point. Based on distribution histograms of the number of ingested particles per cell, thresholds were set based on the cell type and particle type to compare the phagocytic capability. (A) For RAW264.7 macrophages, 15 or more particles per cell (15+) were set for 4.5 μm beads, 50 or more particles per cell (50+) were set for 0.8 μm beads, and 10 or more particles per cell (10+) were set for SRBCs. (B) For BMMs, 10 or more particles per cell (10+) were set for 4.5 μm beads, 15 or more particles per cell (15+) were set for IgG-SRBCs, and 50 or more particles per cell (50+) were set for 0.8 μm beads.

Figure S2. BK is required for large but not small particle ingestion. (A) Representative images showing multiple RAW264.7 cells under conditions indicated. Cells were loaded with 4.5 μm beads and 0.8 μm beads for 60 min. Paxilline reduced the uptake of 4.5 μm beads but not 0.8 μm beads. (B) Dose dependent effect of Paxilline on the ingestion of 4.5 μm beads by RAW264.7 cells. Cells were pre-treated with Paxilline for 30 min. (C) Representative images showing multiple RAW264.7 cells under conditions indicated. Paxilline reduced the uptake of SRBCs by RAW264.7 cells. (D) Paxilline treatment prevented BMMs from ingesting 4.5 μm beads but not 0.8 μm beads. (E) Paxilline treatment dramatically inhibited the uptake of SRBC by BMMs. (F, G) BK KO reduced the ingestion of 4.5 μm beads and SRBC but not 0.8 μm beads.

Figure S3. NS1619, the agonist of BK, increased uptake of 4.5 μm beads and SRBC but not 0.8 μm beads. Cells were pre-treated with NS1619 (20 μM) for 30 min.

Figure S4. Dose dependent effect of NS1619 on the ingestion of 4.5 μm beads in RAW264.7 cells. RAW264.7 cells were treated with different concentration of NS1619 as indicated for 30 min prior to phagocytosis assays and then incubated with opsonized 4.5 μm polystyrene beads at 37°C for 60 min.

Figure S5. Large and small particles occupy distinct pools of vesicle in macrophages. (A) Representative images of RAW264.7 cells loaded with different volume of 0.8 μm beads. (B) Dose dependent uptake of 0.8 μm beads by RAW264.7 cells. X axis represents the volume of beads per well which contains 3 x 10^4 cells. The number of ingested beads per cell were analyzed by dividing the fluorescence intensity of Z stacked confocal images of a whole cell by fluorescence intensity of individual bead using ImageJ software. (C) Neither NS1619 nor PAX had effect on the uptake of 0.8 μm beads when RAW264.7 cells were loaded with saturate dose (60 μL/well) of 0.8 μm beads. Cells were pre-treated with NS1619 (20 μM) or PAX (1 μM) for 30 min. (D) Dose dependent uptake of 4.5 μm beads by RAW264.7 cells. X axis represents the ration of number of beads vs the number of cells. (E) Effects of NS1619 (20 μM) and PAX (1 μM) on the uptake of 4.5μm beads at a moderate dose, i.e. a ratio of 25~50 : 1 (bead : RAW264.7 macrophage) which was used for most experiments. (F) Representative images of RAW264.7 cells co-loaded with 4.5 μm beads and moderate dose (7 uL/well) of 0.8 μm beads. (G) Representative images of RAW264.7 cells co-loaded with 4.5 μm beads and saturate dose of 0.8 μm beads. (H) BK was only recruited to the surface of 4.5 μm beads (asterisks) but not 0.8 μm beads (arrows) at phagocytosis initiation. However, BK was recruited to the surface of both of 4.5 μm beads and 0.8 μm beads at late stage of phagocytosis. RAW264.7 cells expressing BK were co-loaded with 4.5 μm beads and 0.8 μm beads for 5 min (upper) or 45 min (lower).

Figure S6. Lysosomal exocytosis induced by large but not small beads. (A) Large (4.5 μm) beads induced lysosomal exocytosis as indicated by β-hexosaminidase in culture medium. Lysosomal exocytosis were reduced by PAX (1 μM) and increased by NS1619 (20 μM) in RAW264.7 cells. BAPTA-AM (1 μM) inhibited the effect of NS1619 on lysosomal exocytosis. (B) Saturate dose (60 μL/well) of 0.8 μm beads didn't alter the level of β-hexosaminidase in medium.

Figure S1

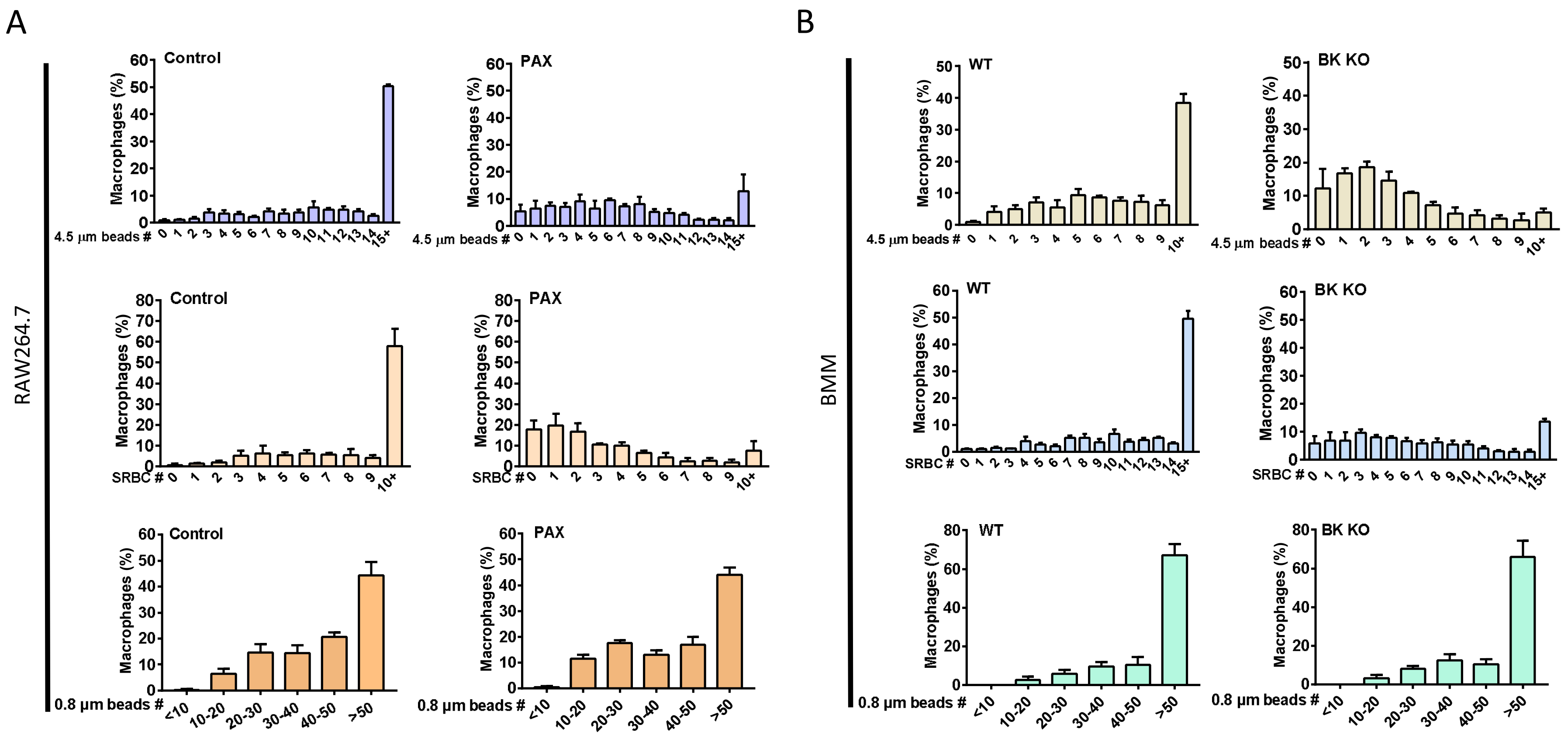

A
B
RAW264.7
BMM
Control
PAX
WT
BK KO
Macrophages (%)
4.5 µm beads #
SRBC #
0.8 µm beads #

Figure S2

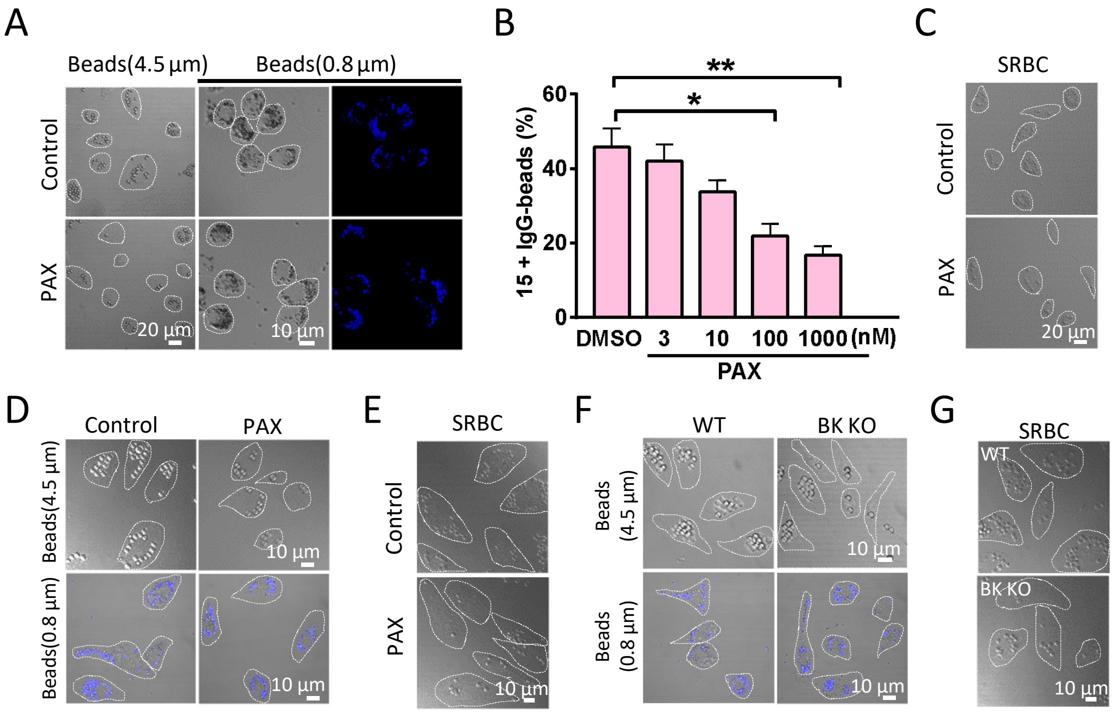

A
Beads(4.5 µm)
Beads(0.8 µm)
Control
PAX
20 µm
10 µm
B
**
*
60
40
20
0
15 + IgG-beads (%)
DMSO
3
10
100
1000
(nM)
PAX
C
SRBC
Control
PAX
20 µm
D
Control
PAX
Beads(4.5 µm)
Beads(0.8 µm)
10 µm
E
SRBC
Control
PAX
10 µm
F
WT
BK KO
Beads (4.5 µm)
Beads (0.8 µm)
10 µm
G
SRBC
WT
BK KO
10 µm

Figure S3

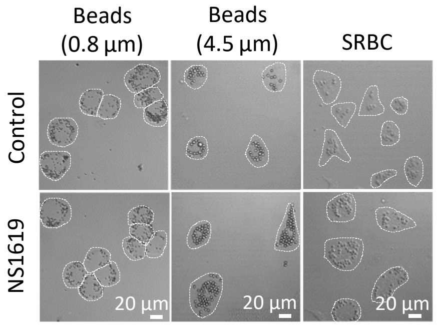

Figure S4

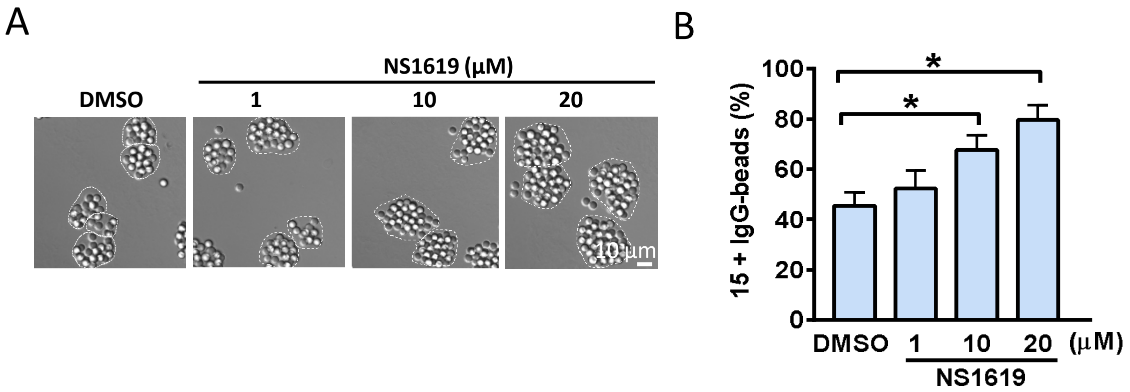
A
NS1619 (μM)
DMSO
1
10
20
10 μm
B
15 + IgG-beads (%)
100
80
60
40
20
0
*
*
DMSO
1
10
20
(μM)
NS1619

Figure S5

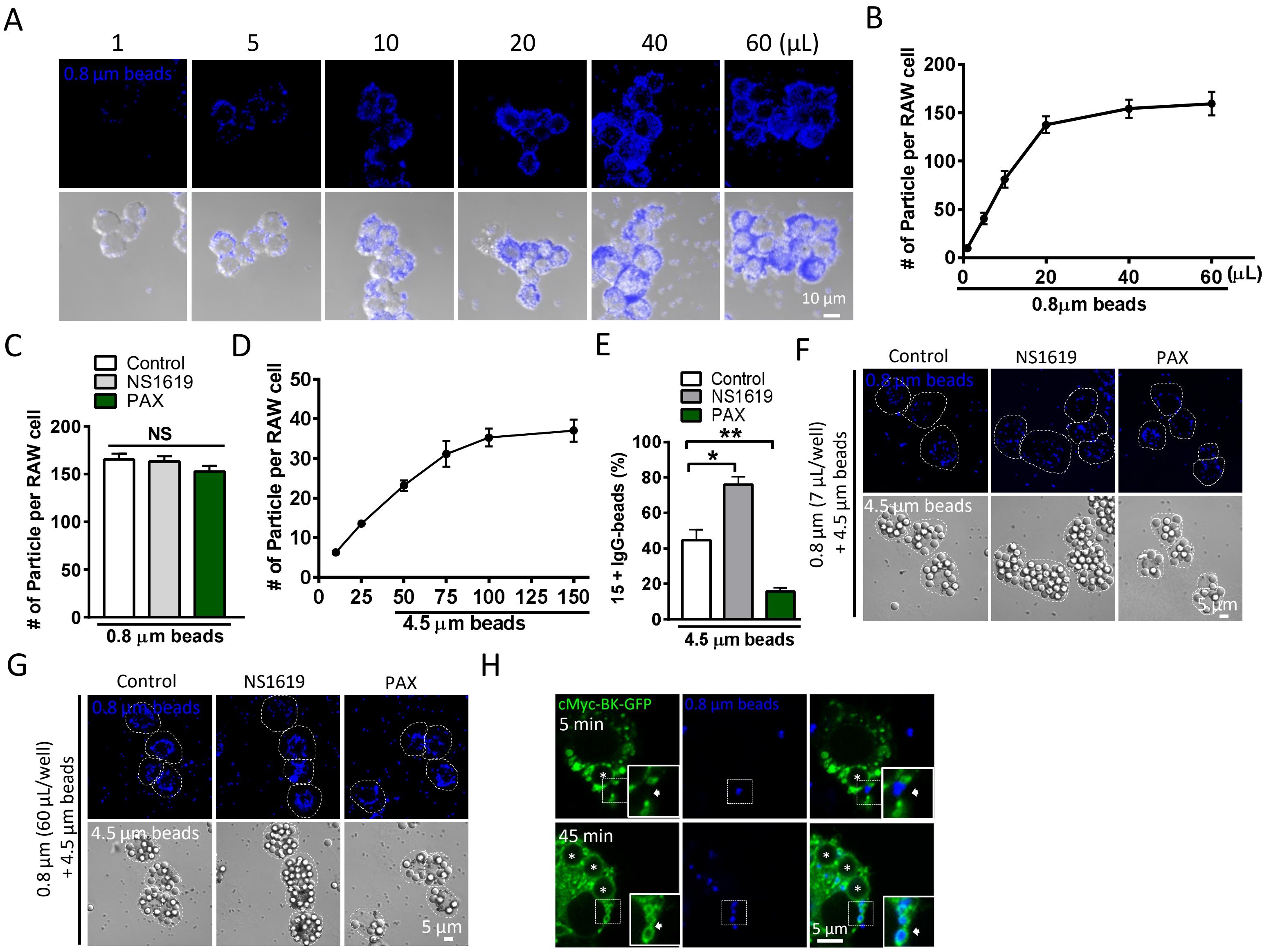

A
1
5
10
20
40
60 (μL)
0.8 μm beads
10 μm
B
# of Particle per RAW cell
200
150
100
50
0
0
20
40
60 (μL)
0.8μm beads
C
Control
NS1619
PAX
NS
# of Particle per RAW cell
200
150
100
50
0
0.8 μm beads
D
# of Particle per RAW cell
50
40
30
20
10
0
0
25
50
75
100
125
150
4.5 μm beads
E
Control
NS1619
PAX
**
*
15 + IgG-beads (%)
100
80
60
40
20
0
4.5 μm beads
F
Control
NS1619
PAX
0.8 μm beads
4.5 μm beads
0.8 μm (7 μL/well) + 4.5 μm beads
5 μm
G
Control
NS1619
PAX
0.8 μm beads
4.5 μm beads
0.8 μm (60 μL/well) + 4.5 μm beads
5 μm
H
cMyc-BK-GFP
5 min
0.8 μm beads
45 min
5 μm

Figure S6

A

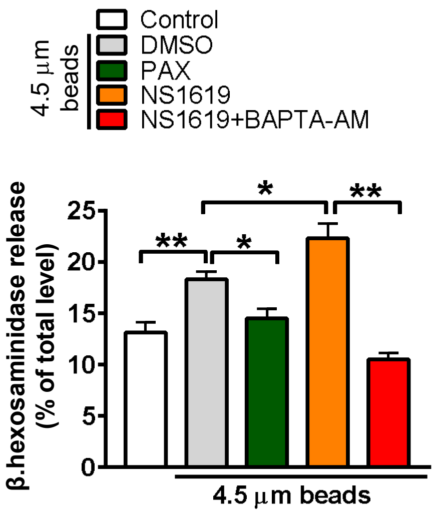

Control
DMSO
PAX
NS1619
NS1619+BAPTA-AM
4.5 μm beads
β.hexosaminidase release (% of total level)
25
20
15
10
5
0
**
*
*
**
4.5 μm beads

B

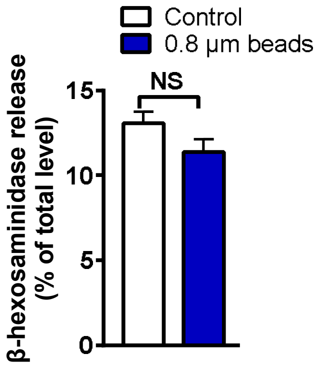

Control
0.8 μm beads
β-hexosaminidase release (% of total level)
15
10
5
0
NS
